# A narrative sequencing and mentalizing training for adults with autism: A pilot study

**DOI:** 10.3389/fnbeh.2022.941272

**Published:** 2022-08-18

**Authors:** Tom Bylemans, Elien Heleven, Kris Baetens, Natacha Deroost, Chris Baeken, Frank Van Overwalle

**Affiliations:** ^1^Brain Body and Cognition, Department of Psychology, Center for Neuroscience, Vrije Universiteit Brussels, Brussels, Belgium; ^2^Ghent Experimental Psychiatry (GHEP) Lab, Department of Head and Skin (UZGent), Ghent University, Ghent, Belgium; ^3^Department of Psychiatry, University Hospital (UZ Brussel), Vrije Universiteit Brussel (VUB), Brussels, Belgium; ^4^Department of Electrical Engineering, Eindhoven University of Technology, Eindhoven, Netherlands

**Keywords:** autism, training, cerebellum, sequencing, mentalizing, narrative coherence, adults

## Abstract

Adults diagnosed with autism experience difficulties with understanding the mental states of others, or themselves (mentalizing) and with adequately sequencing personal stories (narrative coherence). Given that the posterior cerebellum is implicated in both skills, as well as in the etiology of autism, we developed a narrative sequencing and mentalizing training for autistic adults. Participants with an official autism diagnosis were randomly assigned to a Training group (*n* = 17) or a waiting-list Control group (*n* = 15). The Training group took part in six weekly sessions in groups of three participants lasting each about 60 min. During training, participants had to (re)tell stories from the perspective of the original storyteller and answer questions that required mentalizing. We found significant improvements in mentalizing about others’ beliefs and in narrative coherence for the Training group compared to the Control group immediately after the training compared to before the training. Almost all participants from the Training group expressed beneficial effects of the training on their mood and half of the participants reported positive effects on their self-confidence in social situations. All participants recommended the current training to others. Results are discussed in light of cerebellar theories on sequencing of social actions during mentalizing. Further improvements to the program are suggested. Our results highlight the potential clinical utility of adopting a neuroscience-informed approach to developing novel therapeutic interventions for autistic populations.

## Introduction

As social beings, we continuously share experiences through narratives–causally connected events and actions that are often goal-driven, embedded in temporal sequences, and which allow us to imbue personal experiences with meaning (Trabasso et al., 1992; Capps et al., 2000). Narratives that are coherently construed enable listeners to infer mental states (such as emotions, beliefs, and intentions) that are associated with personal experiences (Lind et al., 2020). The ability to infer such mental states is referred to as mentalizing (Van Overwalle, 2009; Schurz et al., 2014). Autism is a lifelong neurodevelopmental condition characterized by social and communicative difficulties (American Psychiatric Association, 2013; Murphy et al., 2016), which involve difficulties with structuring personal stories (narrative coherence) and mentalizing. Even though autism is a lifelong diagnosis with increasing prevalence rates for adults (Baxter et al., 2015; Brugha et al., 2016), autism research remains largely focused on children (Kasari et al., 2014). Therefore, autistic adults are often unable to find proper evidence-based adult-specific services (Murphy et al., 2011; Howlin and Moss, 2012; Hahler and Elsabbagh, 2015; Vogan et al., 2016; Calleja et al., 2019). The few therapeutic programs that currently exist for autistic adults do not always yield the expected results (Gerhardt and Lainer, 2011; Nicolaidis et al., 2015; Dyrda et al., 2020). To the best of our knowledge, such programs are currently not based on neuroscientific insights. To remedy this neglect, we designed a novel narrative sequencing and mentalizing training for adults based on recent neuroscientific insights on the cerebellum to improve (a) mental state attribution of self and others and (b) coherent storytelling.

The design of the training program was based on recent neuroscientific insights on sequencing functions of the posterior cerebellum implicated in many non-motor behaviors such as language (De Smet et al., 2007; Mariën et al., 2014) and social cognition, including autobiographic memory and the chronological order of social events (Van Overwalle et al., 2014, 2019a; Heleven et al., 2021). Structural and functional deficits in the posterior cerebellum are responsible for most of the social and communicative difficulties observed in autism (D’Mello et al., 2015; Hampson and Blatt, 2015). They are key to autism development (Fatemi et al., 2012; Rogers et al., 2013; Wang et al., 2014; D’Mello and Stoodley, 2015; Sathyanesan et al., 2019) and are still observed in adulthood (Vargas et al., 2005; Hallahan et al., 2009). Furthermore, research has revealed atypical (e.g., hyper- or hypoconnectivity) cerebello-cortical connectivity in autism (Khan et al., 2015; Crippa et al., 2016; Ramos et al., 2019), which has been replicated in adult populations as well (Catani et al., 2008; Olivito et al., 2018).

According to the sequence detection hypothesis (Leggio and Molinari, 2015), the cerebellum functions by identifying temporal sequences in human behavior, automatizing them after repeated exposure leading to smooth behavior, and sending feedback signals to the neocortex when unexpected violations of these sequences occur. A similar cerebellar sequencing function has been suggested for social mentalizing (Van Overwalle et al., 2014, 2019a; Heleven et al., 2019). For example, to recognize a sarcastic remark, one needs to remember the relevant contextual information based on automatized event schemas, recognize a sudden, unexpected change in body language and in intonation, and evaluate verbal comments as reflecting inappropriate judgments of people, exaggerated statements, or non-literal statements. All these social inputs are happening in a chronological sequence. Understanding this sequence is a process subserved by cerebellar sequencing functions–a necessary process that leads to the recognition of social signals and requires mentalizing about the intentions or beliefs behind these social cues. This idea has been corroborated by a variety of sequence-based mentalizing tasks (e.g., Pu et al., 2020; Haihambo et al., 2021; Heleven et al., 2021; Li et al., 2021; Ma et al., 2021a,b). Several connectivity studies have uncovered bidirectional connections between the cerebellum and neocortical mentalizing areas which constitute the larger part of the default mode network (Habas et al., 2009; Krienen and Buckner, 2009; Buckner et al., 2011; Van Overwalle and Mariën, 2016; Van Overwalle et al., 2019b,^2020^). This confirms the close neural synchrony between the cerebellum and neocortex required for identifying sequences in social input and sending feedback signals.

As narratives consist of a temporal sequence of causally connected events, the sequence detection hypothesis also applies to narratives. Some researchers have detected cerebellar activation during narrative processing (Xu et al., 2005; AbdulSabur et al., 2014) and during the integration of narrative (movie) events into coherent event sequences (Lahnakoski et al., 2017). Importantly, areas that are involved in narrative production (Mar, 2004) are also connected to the cerebellum (Blatt et al., 2013; Watson et al., 2014; Palesi et al., 2017). Over- and underconnectivity between these areas has been observed in autism (Khan et al., 2015; Igelström et al., 2017; Olivito et al., 2018).

Both mentalizing and narrative coherence are social skills that remain difficult for autistic adults. First, evidence has shown continued mentalizing difficulties (Chung et al., 2014), such as problems with advanced mentalizing tasks that include non-literal expressions such as sarcasm and irony (Happé, 1994; Jolliffe and Baron-Cohen, 1999; Martinez et al., 2019; Morrison et al., 2019). Most of the difficulties are experienced during implicit (i.e., fast and automatic) mentalizing (Schuwerk et al., 2014), as has been observed in naturalistic tasks (Yoshida et al., 2010; Rosenblau et al., 2015) and in studies using eye-gaze tracking (Senju et al., 2009; Kirchner et al., 2011; Schneider et al., 2013). Some researchers have pointed to issues with explicit (i.e., reflective and conscious) mentalizing as well (Cole et al., 2018), however, these results are less clear-cut. Autistic adults are also often less fluent in reflective functioning and social metacognition (i.e., understanding your own mental states during interaction with others) (Roeyers and Demurie, 2010; Grainger et al., 2014)–important components of self-focused mentalizing.

Second, researchers have observed both macrostructural and microstructural narrative incoherence in autistic adults (McCabe et al., 2013; Geelhand et al., 2020). Macrostructure refers to overall narrative structure such as the temporal-causal order, while microstructure refers to internal linguistic elements such as transition words (e.g., “therefore”), cohesive devices (e.g., conjunctions such as “and”), and grammatical/syntactical organization. Autistic adults tend to focus more on non-essential details while narrating events (Barnes and Baron-Cohen, 2012), inaccurately use transition words (Colle et al., 2008), and tend to make less use of cohesive devices (McCabe et al., 2013).

In the current training program, we looked at narrative coherence from a multidimensional perspective as proposed by Reese et al. (2011). According to this multidimensional model, narrative coherence can be subdivided into *contextual* coherence (i.e., when and where of the events), *chronological* coherence (i.e., temporally sequenced events), and *thematic* coherence (i.e., personal reflections and affective evaluations). Although research on autism using this model is non-existent, there is evidence pointing to a relationship with mentalizing. In general, coherent narratives have been related to improved mentalizing of the self and vice versa (Köber et al., 2018; Reese et al., 2011). More specifically, thematic coherence is associated with self-reflection through providing meaning to narrated events (Köber et al., 2018). Contextual coherence is related to perspective-taking (Baron-Cohen, 2001; Reese et al., 2011)–another subcomponent of mentalizing (Frith and Frith, 2006)–which allows a narrator to understand what the listener knows and doesn’t know. Chronological coherence is related to autobiographical memory because personal narratives are reproductions of our own memories. These autobiographical memories are reduced and less specific in autistic adults (Hare et al., 2007; Lind and Bowler, 2010; McDonnell et al., 2017; Anger et al., 2019). Research has furthermore shown a strong overlap between neural systems for mentalizing and for autobiographical memory, as they are all part of the default mode network in the neocortex and cerebellum (Andrews-Hanna et al., 2014; Van Overwalle et al., 2014).

In sum, recent cerebellar findings demonstrated that mentalizing and narrative coherence are strongly interrelated capacities, and are both subserved by cerebellar sequencing functions. Moreover, cerebellar pathology is a key etiological factor in autism. Inspired by these findings, we developed a sequencing-based narrative mentalizing training for autistic adults. The training program aimed at improving self- and other-mentalizing and narrative coherence by asking participants to repeatedly (re)tell narratives. All training activities were inspired by prior theoretical and clinical work on narrative production and mentalizing. We selected elements of these earlier programs that were in line with the sequencing function of the cerebellum, including:

(a) using story grammar elements and visual aids to help structure the temporal order of stories (Moreau and Fidrych, 1994; Spencer and Slocum, 2010; Petersen et al., 2014),
(b) re-telling narratives and combining this with perspective-taking (García-Perez et al., 2008; Spencer and Slocum, 2010; Dodd et al., 2011; Petersen et al., 2014),
(c) focusing on causal relations within stories (Gillam et al., 2015),
(d) engaging in conversations that involve understanding the mental states of other participants and story protagonists by asking and answering story-based questions (Cavallini et al., 2015), and
(e) metacognitive reflection and perspective-taking conversations between participants (Trautwein et al., 2020).

All elements were adapted to an adult population. The current training strongly differs from this earlier work by relying on sound neuroscientific theories for the selection of training activities and by acknowledging the important relationship between mentalizing and narrative coherence.

Based on the sequence detection hypothesis (Leggio and Molinari, 2015), we hypothesized that repeatedly exposing participants to sequence-related narratives and mentalizing inferences during narratives, will improve narrative coherence and mentalizing capacities. We expected significant improvements in these skills in a Training group compared to a waiting-list Control group. Since the major focus of our program was to improve the sequential representation of narratives and embedment of thoughts and emotions within narratives, we expected most improvements on the coherence dimensions reflecting these skills, namely, the chronological and thematic dimensions, respectively.

## Materials and methods

### Participants

High-functioning autistic adults (i.e., with an average to high intelligence level) were recruited through several non-profit autism organizations, diagnostic centers, and autism coaches. Flyers were distributed through social media. A total of 32 participants (17 female, 15 male) with an age range from 18 to 62 years (*M* = 37.06, *SD* = 12.56) replied to the call and were all included in this study. A total of 17 participants were assigned to the Training group (9 female, 8 male; Mean age = 36.35 years, *SD* = 11.43), 15 participants were assigned to the Control group (8 female, 7 male; Mean age = 37.87 years, *SD* = 14.11). To make sure that age and gender were equally distributed across groups, for each new participant, assignment was determined on the sample characteristics (i.e., age and gender) of the already existing groups. When sample characteristics of both groups were balanced, new participants were randomly assigned.

Participants were officially diagnosed with Autism Spectrum Disorder (ASD) by independent multidisciplinary teams at several diagnostic centers based in Belgium. All participants provided proof of their diagnosis by sharing official documents with the primary investigator during a home visit (see section “Procedure”). All participants were free of concurrent neurological diagnoses or comorbid psychotic disorders. This was ascertained through the general questionnaire (see section “Measure”) and by interviewing the participants during the home visit. Other comorbid psychiatric disorders such as attention-deficit hyperactivity-disorder (ADHD), obsessive-compulsive disorder (OCD), anxiety, and depression, were accepted as such comorbidities are difficult to exclude from an autistic sample (Damiano et al., 2014). All participants received 20 euros for the completion of the pre-training testing and 20 euros for the completion of the post-training testing. Participants signed a written informed consent after being informed of the details of the study. This study was approved by the medical ethical committee of the University Hospital Brussels, in line with the guidelines of the Declaration of Helsinki (2013).

Two participants dropped from the training program prior to starting the sessions due to unexpected schoolwork. Three other participants ended the training prematurely (i.e., after four sessions) because of a failure to find overlapping dates for the last two training sessions. Two of them completed a post-training test. All (incomplete) data of these five participants have been included as input in the analyses.

### Procedure

**FIGURE 1:**
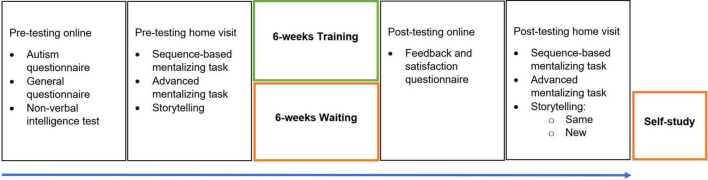
Study Procedure. Pre-testing consisted of online questionnaires and intelligence test, and a home-visit. Participants in the Training group immediately started their 6-week training after pre-testing while participants in the Control group waited. Post-testing consisted of an online feedback questionnaire and a home visit. Participants in the Control group received a self-study bundle of the training.

Due to COVID 19 restrictions at the time of the study, the entire program was organized online, albeit with live interactions between participants and the experimenter. A graphic overview of the procedure is shown in Figure 1. After confirming participation in the study, participants signed the informed consent and completed several online questionnaires and a non-verbal intelligence test. Participants received a document and a video with detailed information about the training procedure. We attempted to attract and motivate potential participants by providing full transparency and maximal predictability. After completing the questionnaires and non-verbal intelligence test, participants were contacted again for a home visit. During this visit, under the supervision of the experimenter, participants completed two sequencing-based mentalizing tasks on a tablet, told two stories, and were administered an advanced mentalizing task. Further details on these measures are provided below. The participants followed each session of the training in small groups of three people (always the same group). They were invited to complete an online agenda in order to pinpoint dates for weekly training sessions. The total training for each participant was about 6 h, not including homework assignments and pre- and post-testing. Data collection started in February 2021 and ended in February 2022.

Upon completion of the training program, participants were invited to fill in a feedback and satisfaction questionnaire. Participants again completed the two mentalizing tasks on a tablet during a home visit, told the two pre-training stories again [i.e., the same topic, henceforth referred to as *Post (same) narratives*], told two new narratives [henceforth referred to as *Post (new) narratives*], and completed the advanced mentalizing task. Participants received feedback on their pre-training results. Importantly, the time between the first and second assessment was the same for both groups, between 6 and 7 weeks.

The Control group did not participate in any form of training or specific activities during their waiting period. They received the training in a printed self-study version after the second assessment, in which all training information was carefully printed and exercises were accompanied with blank lines to be completed by the participants.

### Training program

The program focused on improving mentalizing and narrative sequencing. Mentalizing was trained by retelling narratives from the original storyteller’s perspective while attending to the thoughts and emotions embedded in the overall storyline. Questions were asked that pertained to the mental states embedded within the story. Learning was further stimulated through verbal reinforcement. Narrative coherence was trained through mini lessons focusing on story grammar elements (i.e., setting, character, initiating event, problem, thoughts, emotions, actions, consequences, and resolution). Participants were visually aided by icons that depicted these elements and were reminded to use transition words. These visual aids were gradually removed to increase the internalization of applying story elements.

The program consisted of six sessions which were subdivided into two main parts; the first three sessions focused on theory (mini lessons; psychoeducation, tips and tricks, and impact of poorer mentalizing and narrative coherence) and retelling prewritten narratives, while the last three sessions focused on generating and retelling personally experienced narratives (see Table 1). After each session, participants received homework that was discussed at the beginning of the next session. For each session, an extensive manual was written to ensure consistency both between and within groups. In general, the main investigator followed an amount of pre-determined steps which can be consulted in Table 2. More detailed information on the training (i.e., specifics of each session and deviations from the general steps outlined in Table 2) is provided in the Supplementary material. We used SAFE recommendations to maximize training effects (Durlack et al., 2011)–including a *sequenced*, step-by-step training approach, *active* learning, *focusing* sufficient time on skill development, and having *explicit* learning goals.

**TABLE 1:** Specific topics of each session and mini-lesson.

Session theme	Mini-lesson
*1. Retelling stories with visual aids*	*Story structure*
*2. Retelling stories with visual aids and transition words*	*Transition words*
*3. Retelling stories without visual aids*	*Mentalizing*
*4. Generating stories with visual aids and prompts*	*Summary of previous mini-lessons*
*5. Generating stories through preparations at home*	*None*
*6. Generating stories spontaneously*	*None*

Schematic overview of the training sessions. The full manual can be requested from the main author of the study.

**TABLE 2:** Steps followed in each session.

1. Brief introduction
2. Discussion of homework assignment
3. Mini-lesson (first four sessions)
4. Listen to story or generate story
5. Retell story
6. Questions
7. Reflection on session
8. Explanation of homework assignment

### Measures

As noted earlier, some measures were administered online due to COVID restrictions.

#### General questionnaire

Participants completed an online general questionnaire which inquired about previous psychological and medical interventions, medical and sleeping problems, caffeine and alcohol intake, drug use, employment status, and educational background.

#### Autism questionnaire and social responsiveness scale for adults

Both self-report questionnaires were administered online and were included as covariates in the analyses to counteract potential confounding influences of a heterogeneous sample. An AQ score above 32 served as a cut-off for autism traits (Baron-Cohen et al., 2006). The SRS-a additionally identified the severity of social difficulties related to autism. An SRS-a score above 75 predicts (severe) autism traits (Bölte et al., 2011).

#### Raven progressive matrices

The RPM (Raven and Court, 1998)–a non-verbal intelligence test–measured deductive reasoning and fluid intelligence. The RPM was administered online. An advantage of online RPM administration is that is avoids potential confounds such as stress and concentration problems due to a novel, potentially overstimulating test environment. Research has previously shown a positive correlation between RPM grades and standard IQ scores (O’Leary et al., 1991). Participants saw 60 incomplete symbol patterns and had to select the completing part. The RPM was administered to ensure at least average intelligence in the sample and preferred to conventional intelligence tests which can underestimate intelligence in autistic individuals (Dawson et al., 2007). Scores on the RPM were compared to norms collected in Great Britain (1992) based on a sample of 629 adults (Raven and Court, 1998).

#### Narrative coherence coding scheme

The NaCCS (Reese et al., 2011) was used to examine improvements in narrative coherence. At pre-test, participants told one story about their most positive and most negative life experiences (counterbalanced). We specifically elicited a positively and negatively valenced narrative because previous research has revealed that negative narratives are often more coherent than positive narratives (Fivush et al., 2008; Vanderveren et al., 2019). We, therefore, wanted to make sure that every participant told a story with a positive and a negative valence. This way, we could also explore whether the training would be able to improve global coherence or rather valence-specific coherence. At post-test, the topics of the stories at pre-test were retold [*Post (same) narratives*]. In addition, they told two completely new stories [*Post (new) narratives*]. Participants were explicitly asked for highly emotionally valenced narratives as this provided the highest chance of detecting any thematic elements. Participants could tell as long or short a story as they preferred while the investigator would passively listen. Narratives were recorded on a Sony audio recorder and transcribed for further analysis. Two raters (including the main investigator TB) coded the narratives on the 3 dimensions of the NaCCS as suggested by Reese et al. (2011); (a) Contextual coherence (i.e., when and where of the events), (b) chronological coherence (i.e., events sequenced on a narrative timeline), and (c) thematic coherence (i.e., self-focused mentalizing involving personal reflections and affective evaluations). Each dimension was scored on a scale from 0 to 3, and the overall score on a scale from 0 to 9.

First, 30% of all the narratives were coded by the two raters after which inter-rater reliability was assessed by calculating Intraclass Correlation (ICC) as in previous research (e.g., Chen et al., 2012; Sales et al., 2013; Vanaken et al., 2021). We also calculated Krippendorff’s alpha, a stricter reliability coefficient. Because inter-rater reliability was initially low and the main investigator was not blind to group assignment, 30% of all the stories were recoded by 3 trained, independent blinded raters and compared to the main investigator’s initial scores. As inter-rater reliability was sufficient to high (Chronological, *ICC* = 0.86, α = 0.73; Contextual, *ICC* = 0.93, α = 0.80; Thematic, *ICC* = 0.87, α = 0.70), ratings of the main investigator were kept as input for the final data analysis.

#### Verbal and pictorial sequencing tasks

**FIGURE 2:**
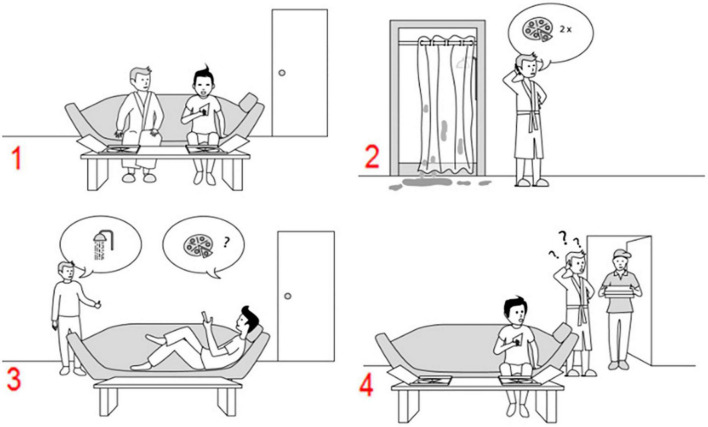
An example of a false belief sequence in the Pictorial Sequencing Task (the correct order is 3–2–1–4). Participants have to select, in the correct order, the first picture on the screen, then the second picture, and so on. Each time, the pictures move in the order indicated by the participant (Heleven et al., 2019).

To explore the program’s effectiveness, we used recently developed sequencing tasks (Heleven et al., 2019) which measure (other-focused) mentalizing in a sequential context. The recruitment of the cerebellum in these tasks has been validated with fMRI research (Heleven et al., 2019), in cerebellar patients (Van Overwalle et al., 2019a), and with cerebellar neurostimulation (Heleven et al., 2021). Participants had to generate the correct chronological order of four scrambled pictures or sentences. The pictural and verbal versions of the task were presented in a counterbalanced order across participants. Two sets of stimuli were created for each version to allow pre- to post-testing. Because we were only interested in training effects on sequence-based mentalizing, we limited our analyses to false- and true- belief scenarios which require mentalizing about another person’s belief in order to generate the correct chronological order (Heleven et al., 2019). In false-belief scenarios, a false or outdated belief occurs due to an unobserved change in reality as experienced from the perspective of a protagonist, while in true-belief scenarios, such a change in reality was observed. See Figure 2 for an example of a pictorial false belief scenario.

The sequencing tasks were administered via a Microsoft Surface Pro tablet with an attached keyboard and run on E-prime 3 software (Psychology Software Tools, Inc, 2016, Pittsburgh, PA, United States). Participants viewed the pictures/sentences on the screen with a number from 1 to 4 in front of each stimulus. Responses were given with the numerical keys on the keyboard to select the first, second, third, and fourth picture/sentence in the sequence. After the third picture/sentence was selected, participants could restart the trial if they had made a mistake, or confirm their chosen order and continue to the next trial. Participants were asked to respond as accurately and as fast as possible. As high-functioning autistic adults have been known to pass false-belief tasks (Channon et al., 2014; Eddy, 2019), we expected ceiling effects for the accuracies of all scenarios. However, as autistic people often use compensatory strategies when time permits (Livingston et al., 2020), and given that such strategies are often inefficient and time-consuming (Livingston and Happé, 2017), we did expect changes regarding reaction times. All mean data, including reaction times and accuracies, can be consulted in the Supplementary material.

#### Animated triangles task

As some high-functioning autistic adults can pass certain false-belief tasks, we decided to administer the ATT (Abell et al., 2000) as well–an advanced (other-focused) mentalizing task in which participants watch nine short movie clips of moving triangles. For example, in one video, a small triangle is ostensibly trying to *persuade* a big triangle to move away from an exit, although there is no sound nor human movement to imply this, only the movements of the triangles. In this sequence, participants mentalize when they understand that the small triangle is trying to change the mental state of the big triangle (i.e., persuade the big triangle to move away). The videos were presented on a Microsoft Surface Pro tablet. Participants were asked to describe what they had seen immediately at the end of each clip. For the current study, we looked at verbal descriptions of the mentalizing movement sequences in which an interaction occurs between the triangles, and mental state appreciation is required to understand the sequence. Verbal descriptions were recorded on a small Sony audio recorder and transcribed for further analysis. Two raters scored each description on two dimensions; (1) Intentionality (i.e., use of mental state verbs to describe the intentional nature of the interactions) with a score between 0 and 5 for each video, and (2) appropriateness (i.e., understanding the meaning of the depicted sequence) with a score between 0 and 2 for each video. First, 30% of all the descriptions were scored by the two raters after which inter-rater reliability was assessed again by Intraclass Correlation (ICC) and Krippendorff’s alpha. Inter-rater reliability for both dimensions was sufficient to high (Appropriateness; *ICC* = 0.96, α = 0.67; Intentionality; *ICC* = 0.67, α = 0.93). After recoding these 30% verbal descriptions through consensus, the remaining 70% were coded by the main investigator.

#### Feedback and satisfaction questionnaire

We developed our own questionnaire to gather qualitative feedback on the training program. Participants rated their degree of satisfaction on a Likert Scale ranging from 1 (very unsatisfied) to 5 (very satisfied). Rated aspects included; Therapist, general training format, instructions, exercises, training length, training goals, personal benefits, training moments, general organization, training difficulty, and training content. Participants could elaborate on their ratings and could recommend improvements to the training program. Participants were further asked yes/no questions pertaining to: the likeliness of recommending this training to others, whether they felt that these kinds of training programs were currently lacking, whether or not they felt that the training was effective/helpful to overcome some of their daily difficulties, and whether or not the training had a positive effect on their mood and social self-confidence. Finally, we asked yes/no questions about their motivation and concentration levels during the training sessions.

### Data analysis

We conducted several repeated measures MANCOVA’s with total AQ and SRS scores as covariates to control for heterogeneity in our sample. Missing data and outliers were replaced by *Sampling Mean Estimation*. An overview of missing data can be consulted in the Supplementary material. Outliers were visually detected through boxplots. If, after Sampling Mean Estimation, outliers were still detected in the boxplots, we logarithmically transformed the data prior to analysis. Note that the logarithmic transformation was not intended to rectify potential non-normal distribution of the data. For all our data, normality was verified.

We compared pre-training scores between the Training and the waiting-list Control group to ensure that they started at the same level. Pre- and Post-training scores were compared within and between groups, and Bonferroni-corrected. The waiting-list Control group did not receive any intervention between pre- and post-testing.

## Results

### Autism traits and intelligence

See Table 3 for an overview of average AQ and SRS scores, and proportions of RPM grades for both groups. RPM scores indicated that our sample consisted of high-functioning autistic adults.

**TABLE 3:** Autism questionnaire (AQ), SRS, and RPM scores.

	**Training group**	**Control group**
Total AQ: mean (SD)	31.65 (*7.79*)	32.67 (*9.46*)
Total SRS: mean (SD)	92.65 (*24.25*)	93.40 (*36.17*)
RPM IV/V (Below-average intelligence):% (number of participants)	0% (0)	14% (2)
RPM III (average intelligence):% (number of participants)	59% (10)	64% (10)
RPM II/I (Above-average intelligence):% (number of participants)	41% (7)	21% (3)

Total AQ and SRS scores were used as covariates in the analyses to control for the heterogeneity in our sample. Only two participants scored low on the RPM. However, both participants shared their IQ scores on official diagnostic reports (one average and one above-average) and both received academic diplomas. We therefore included them in our sample of high-functioning autistic adults.

### Narrative coherence

**FIGURE 3:**
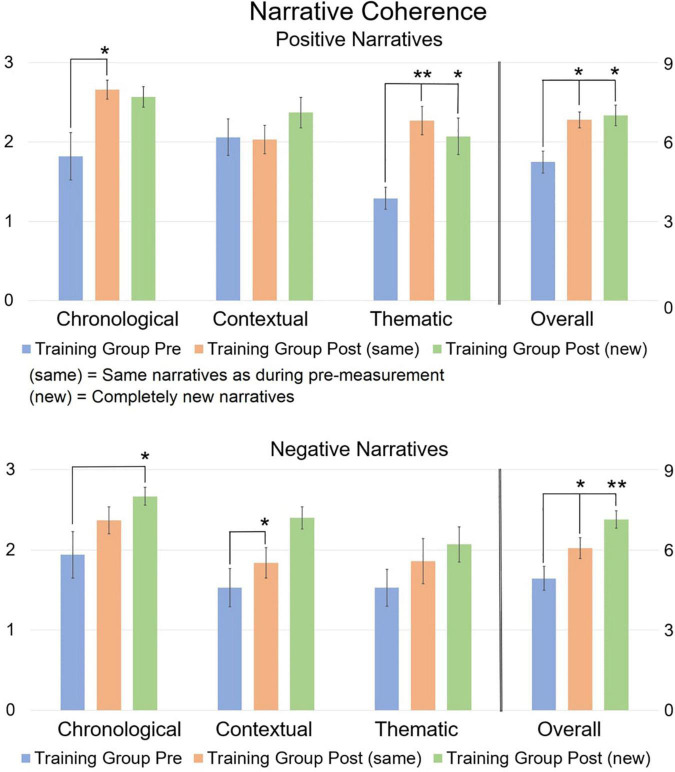
Mean narrative coherence scores comparing pre and post scores. The 0–3 scale on the left refers to the three distinct coherence dimensions, while the 0–9 scale on the right refers to the overall coherence. The post-measurement refers to narratives that are the “same” or “new” compared to the pre-measurement. Error bars represent the Standard Error of Means (SEM). *p < 0.05, **p < 0.001 *F*-test between pre- and post-measurement. Asterisks refer to the comparison between pre vs. post (new) and between pre vs. post (same), and not between post (new) and post (same).

Given the distinct post-measures (same or new), we ran separate Repeated Measures MANCOVAs with Time (pre vs. post) and Group (training vs. control) on the pre-training measure and each distinct post-training measure. None of the covariates showed an interaction with any of the measures, indicating that none of the covariates mediated the observed effects. See Figure 3 for a visual overview of the data. Means and standard deviations for each dimension, as well as an additional Figure directly comparing scores between groups, can be consulted in the Supplementary material.

Repeated Measures MANCOVA revealed a significant multivariate interaction of Time × Group [*F*_(8,21)_ = 2.9, *p* = 0.024, η^2^ = 0.525] for pre-training and post (same) narratives. At pre-training, there were no significant differences between groups. At post-training, simple main effects analysis revealed a significant difference between the Training group and the Control group for chronological coherence [*t*(31) = 4.13, *p* < 0.001] and overall coherence [*t*(31) = 3.23, *p* = 0.003] of positively valenced narratives. When comparing pre-training vs. post (same) narratives within groups, a significant improvement was found for the Training group on chronological coherence [*t*(16) = 2.82, *p* = 0.009], thematic coherence [*t*(16) = 5.19, *p* < 0.001], and overall coherence [*t*(16) = 3.38, *p* = 0.002] for positively valenced narratives, and on overall coherence for negatively valenced narratives [*t*(16) = 2.11, *p* = 0.044]. No significant differences were found for the Control group.

Repeated Measures MANCOVA also revealed a significant multivariate interaction of Time × Group [*F*_(8,21)_ = 2.742, *p* = 0.03, η^2^ = 0.511] for pre-training narratives and post (new) narratives. At pre-training, simple effects analysis revealed no significant difference between both groups, except for thematic coherence of positively valenced narratives which was higher in the Control group compared to the Training group [*t*(31) = 5.55, *p* < 0.001]. At post-training, simple effects revealed a significant difference between the Training group and the Control group for chronological coherence [*t*(31) = 5.64, *p* < 0.001] and overall coherence [*t*(31) = 3.63, *p* = 0.001] of positively valenced narratives, and for chronological [*t*(31) = 3.82, *p* < 0.001] and overall coherence of negatively valenced narratives [*t*(31) = 2.82, *p* = 0.009]. When comparing pre-training narratives and post (new) narratives within groups, a significant improvement was found for the Training group on thematic coherence [*t*(16) = 3.38, *p* = 0.002; but note that this group had lower thematic scores to begin with compared to the Control group] and overall coherence of positively valenced narratives [*t*(16) = 2.86, *p* = 0.008], and on chronological coherence [*t*(16) = 2.47, *p* = 0.02], contextual coherence [*t*(16) = 3.05, *p* = 0.005], and overall coherence of negatively valenced narratives [*t*(16) = 3.90, *p* < 0.001]. No significant differences were found for the Control group.

### Animated triangles task

Repeated Measures MANCOVA revealed no significant differences in pre- to post-training ATT scores for the Training group compared to the Control group.

### Verbal and pictorial sequencing tasks

**FIGURE 4:**
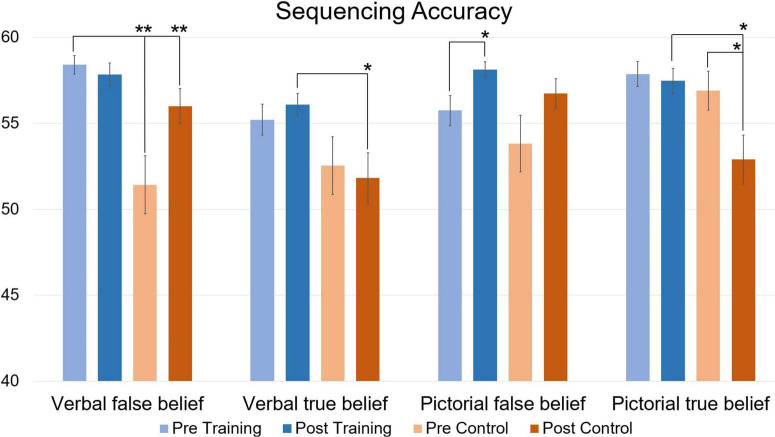
Accuracy of verbal and pictorial true- and false-belief sequencing comparing pre and post scores in function of Training and Control groups, expressed in mean total accuracy scores per condition (maximum score is 60). Error bars represent the Standard Error of Means (SEM). *p < 0.05, **p < 0.001 *F*-test between pre- and post-measurement and between groups.

When looking at accuracy (Figure 4), repeated measures MANCOVA revealed a significant multivariate interaction of Time × Group [*F*_(4,25)_ = 6.086, *p* = 0.001, η^2^ = 0.493]. At pre-training, simple main effects analysis revealed significantly better performance for the Training group compared to the Control group for verbal false belief sequencing [*t*(31) = 4.07 *p* < 0.001]. At post-measurement, these significant differences disappeared, probably due to a significant improvement in performance for the Control group [*t*(14) = 4.22, *p* < 0.001]. At post-testing, simple main effects analysis revealed significantly higher accuracies for the verbal and pictorial true belief sequences in the Training group relative to the Control group [*t*(31) = 2.71, *p* = 0.010, *t*(31) = 2.75, *p* = 0.008, respectively]. Of most importance, relative to baseline pre-testing, the Training group significantly improved on pictorial false belief sequencing [*t*(16) = 2.11, *p* = 0.034], while the Control group performed significantly less well on the pictorial true belief sequences [*t*(14) = −4.00, *p* < 0.001]. Note that the post-testing improvements of the Training group for pictorial false belief sequencing or for (verbal and pictorial) true belief sequencing, were not confounded by better performance at pre-training, where as noted above, simple effects revealed only better performance for verbal false belief sequencing of the Training group compared to the Control group.

Repeated measures MANCOVA on accuracy furthermore revealed a significant multivariate interaction of Time × AQ [*F*_(4,25)_ = 2.844, *p* = 0.018, η^2^ = 0.313], indicating that AQ total scores were associated with the effect of time on performance of the sequencing tasks. Closer inspection of AQ scores did not, however, reveal significant differences between groups on self-reported AQ scores, indicating that differences in AQ scores probably moderated outcomes within groups, but not between groups. A *post-hoc* correlational analysis only revealed a significant positive correlation between AQ scores and accuracy scores for verbal true-belief sequencing at post-test in the Training group (*r* = 0.60, *p* = 0.011).

When looking at response times, Repeated Measures MANCOVA revealed no significant differences in pre- to post-training verbal and pictorial sequencing response times for the Training group in comparison to the Control group.

### Feedback and satisfaction questionnaire

There were 11 out of 12 participants who fully completed the training program and filled in the questionnaire. Table 4 shows an overview of their responses. In general, participants were very satisfied with the training program. Participants reported that the training duration was too short and not challenging enough. Participants liked the transparency of the investigator, the clear structure and repetitive nature of each session, and the duration of each session. Interestingly, with regards to the need and feasibility of future implementations of this training program in healthcare services, and of continued investigation with larger sample sizes, all participants would recommend the training to others and 91% felt that training programs such as these are currently lacking. All participants reported beneficial effects of the training in their daily lives, 91% reported a positive effect on their mood, and 46% reported a positive effect on their self-confidence in social interactions. Motivation remained high throughout the program for all participants.

**TABLE 4:** Feedback and satisfaction questionnaire overview.

Satisfaction	Responses (1 = very unsatisfied, 5 = very satisfied)		
	5	4	≤ 3
Therapist/experiment leader	82%	18%	0%
Training in general	19%	82%	0%
Instructions during sessions	46%	55%	0%
Exercises	27%	73%	0%
Total training duration (amount of sessions)	27%	46%	27%
Length of sessions	27%	73%	0%
Goal of the training	46%	46%	9%
Personal benefits gained from the training	18%	55%	27%
Training organization	4%	55%	0%
Training difficulty level	64%	18%	18%
Training content	46%	55%	0%

**Feedback and Feasibility**	**% yes responses**		**% no responses**

Would you recommend this training to others?	100%		0%
Do you feel that training such as these are lacking?	91%		9%
Was the training helpful for you?	100%		0%
Did you keep your motivation throughout training?	100%		0%
Was it difficult to remain concentrated during the sessions?	18%		82%
Did the training have a positive effect on your mood?	91%		9%
Did the training have a positive effect on your self-confidence for social situations?	46%		55%

## Discussion

### Summary of findings

Social difficulties such as issues with mentalizing and narrative coherence are frequently experienced by autistic adults. To the best of our knowledge, however, existing interventions for this population are not informed by recent neuroscientific advances–a somewhat surprising observation given the strong neurodevelopmental character of autism. For this reason, we proposed a novel narrative sequencing and mentalizing training based on recent neuroscientific insights on sequencing functions subserved by the cerebellum. Participants were repeatedly exposed to narrative sequences. Narratives were (re)told from the perspective of the original storyteller (perspective-taking) and questions were asked that required mentalizing. We hypothesized that repeated exposure to sequences would improve mentalizing and narrative coherence in an autistic Training group compared to an autistic waiting-list Control group.

Autistic adults in the Training group significantly improved on several narrative dimensions. Chronological coherence was improved for both positively and negatively valenced narratives, indicating that participants were able to place narrative events in a more adequate temporal-sequential order after training. Thematic coherence was improved for positively valenced narratives, as participants provided significantly more personal reflections and evaluations after the training program than before, which reflects improved self-focused mentalizing. Contextual coherence only improved for negatively valenced narratives, as participants provided more specific information regarding the time and location of personally experienced events. Overall narrative coherence improved for both positively and negatively valenced narratives. Interestingly, the waiting-list Control group did not improve at post-measurement. In general, we detected improvements in both types of valenced narratives even though prior research suggested that negatively valenced narratives tend to be more coherent than positively valenced narratives (Fivush et al., 2008; Vanderveren et al., 2019). We recommend future studies to further explore valence differences when investigating narratives in autism. For now, we conclude that there is a need for training programs focusing on narrative coherence in autistic samples since both negative and positive narratives can show improvements. Especially given that coherent narratives play an important function in our social lives (Vanaken et al., 2021).

Regarding other-focused mentalizing abilities, the current training program was able to demonstrate improvements of autistic adults in the Training group regarding accuracy of pictorial (non-verbal) false belief sequencing relative to pre-training, and also showed improved accuracy relative to the control group regarding accuracy of verbal and pictorial true belief sequencing. The waiting-list Control group did however also significantly improve in accuracy of verbal false belief sequencing. Although pre- and post-sequencing tasks consisted of different stimuli, improvements might still be due to the mere effect of task repetition. However, we contend that the improvements in the Control group were observed in a different task modality than the Training group (i.e., verbal vs. pictorial sequencing). Although the tasks are related (subserved by sequencing processes), they are also different as non-verbal processing might require different strategies compared to verbal processing. Response times did not significantly improve after training. These are very promising results which demonstrate that the training program has potential to become an effective tool to teaching novel sequencing strategies that enhance mentalizing skills. Future research could compare scores on the sequencing tasks of autistic people after the training to performance by neurotypical individuals in order to investigate whether they achieved a similar performance level. Note that in contrast, accuracy to attribute intentionality to geometric figures moving in human-like (interactive) patterns was not improved. This might be due to the fact that this task does not involve explicitly generating the correct sequence of actions during mentalizing, while the training program focused on improving mentalizing skills combined with sequencing during storytelling. Another explanation might be that geometric figures first need to be anthropomorphized to understand their “social” behavior, while, in contrast, the sequencing tasks clearly deal with people and does not require this extra analytic step, rendering this task perhaps more sensitive to different levels of social sequencing for an autistic population. Other explanations are discussed in the limitations below.

The advancements in mentalizing results are in line with the improvements found for narrative coherence, especially when considering significant improvements for the thematic dimension of narrative coherence (which requires self-focused mentalizing abilities), and the chronological dimension (which requires adequate temporal-sequencing abilities, such as in the sequencing task).

Feedback from participants in the Training group revealed overall satisfaction with the training program. All participants would recommend the current training to others and almost all participants reported a general lack of such programs in current healthcare. All participants reported that the training was helpful. Almost all participants reported beneficial effects of the training on their mood and half of the participants reported positive effects on their self-confidence in social situations. Although these responses cannot be compared against a control group, such feedback is interesting to estimate the feasibility and usefulness of the program. The positive responses of our participants point toward an important need to further develop and investigate the program.

### Limitations and future research

High-functioning adults are generally able to pass simple (false-belief) mentalizing tasks (Channon et al., 2014; Eddy, 2019) but seem to have difficulties with “advanced” mentalizing tasks instead (Murray et al., 2017; Morrison et al., 2019). Such advanced mentalizing tasks often gauge their ability to understand sarcasm, attribute intentionality, detect lies, et cetera. It should be noted that we have not focused on these aspects of mentalizing in the current training program and rather kept a narrower focus.

In light of the cerebellar sequencing function (Leggio and Molinari, 2015), and in line with our hypotheses, we observed accuracy improvements on three out of four belief sequencing conditions. However, this result should be interpreted with some caution as the waiting-list Control group also showed some improvement in mentalizing, albeit in a different condition. In contrast, no improvements were found on the second so-called advanced mentalizing task (the Animated Triangles Task, Abell et al., 2000) in which participants verbally described moving triangles that interacted with each other in human-like patterns. A potential explanation of these non-significant results, noted earlier, is that this task is unrelated to sequencing, which is a fundamental novel aspect of the belief sequencing task. This attests to the importance of including task measures that test the critical novel component of the present training, namely the unfolding of sequences during narration of a story. Moreover, the Animated Triangles Task requires verbal responses which might hamper the sensitivity of detecting mentalizing difficulties in a sample characterized by communication difficulties. Furthermore, research has shown that such “advanced” mentalizing tasks often recruit additional abilities such as executive functions (Zalla and Korman, 2018) and that mentalizing is not always necessarily required to pass the tasks (e.g., Santiesteban et al., 2015). More naturalistic mentalizing tasks (e.g., Rosenblau et al., 2015) or more “advanced” aspects of mentalizing such as recognition of sarcasm, irony, and lie detection could have been more appropriate at detecting mentalizing improvements in the current study.

We should also note that the current sequencing tasks are not exempt from this critique as they likely recruit executive functions as well. Although we cannot exclude the possibility of some effects on executive functions, we assume these additional effects might be minor since we did not detect any improvements in reaction times. In the Supplementary material, we report two types of reaction times: Reaction times between stimulus onset and end of trial (RT-total), and reaction times between stimulus onset and first response (RT1). In previous studies of the sequencing tasks (Heleven et al., 2019), RT1 was considered the most critical processing time, while RT-total might mostly involve processes related to executive functioning, such as remembering, executing, and updating the sequence. Hence, improvements in executive functioning could have led to improvements in total reaction times, for example, through faster remembering of the sequence.

Furthermore, as this was a pilot study, we did not test the potential generalizability of the training to daily life. It has been argued that a lack of generalizability is often a limitation in clinical studies on autism (Carruthers et al., 2020). We are aware that this is a limitation in the current study as well. Note that qualitative feedback from the participants revealed that all participants reported beneficial effects of the training in their daily lives. We admit, however, that these beneficial effects are not necessarily related to improved mentalizing and/or narrative coherence, but could also be due to general training effects such as received empathy and the experience of sharing thoughts with other autistic people. In future developments of this training program, we, therefore, suggest implementing long-term outcomes of training effects, both looking at consistent improvements over time, as well as at effects on daily life functioning. We furthermore suggest implementing methods to increase generalizability. Such methods could include the use of material that is naturalistic and directly applicable to a participant’s personal life, as well as reinforcement of spontaneously occurring generalizations (see Stokes and Osnes, 1989).

Considering the cerebellar sequencing function (Leggio and Molinari, 2015), results regarding narrative coherence are in line with our hypotheses as well. During the training program, a major focus was placed on chronologically ordering narrative sequences and on embedding thematic elements such as thoughts and emotions within narratives. The contextual dimension (i.e., when and where) was covered to a lesser extent. The results of the current training study are reflective of this procedure, especially for chronological coherence as this dimension was improved both for positively and negatively valenced narratives, showing that temporal-sequencing abilities were strengthened.

Other, more general, limitations to the current training study need to be addressed. First, participants were always tested during home visits, and consequently, testing environments varied substantially between participants. However, this procedure had the benefit of reducing the chance of stress/anxiety during testing because of a safe/familiar testing environment. Our decision to test participants at their own place, was grounded in the belief that getting to know the investigator at their own terrain first would lower potential amounts of stress/anxiety. We believed that this would benefit the training program. In line with this cautionary statement, participants in the Training group got to know the investigator better (2 home visits + 6 sessions) than participants in the waiting-list Control group (only 2 home visits), which could have affected stress levels during post-training testing, and thus constitute a potential confound of the results. Future research could, for example, send a second, independent investigator to do the second testing in order to remove this confound (i.e., stress levels should remain the same at both testing times). Another potential option is to provide an equal amount of social attention to both groups (e.g., LaFave et al., 2019). Related to the latter suggestion, a critical limitation of the current study was the lack of participation in any training or activity for the Control group. Future research should provide either an alternative non-sequencing-based program to the Control group or provide means to increase active participation, for example through the organization of group contact without receiving specific training.

Second, it should be noted that we did not inquire about comorbid psychiatric conditions. We only made sure that none of our participants currently, and/or in the past, experienced psychotic or neurologic disorders. We included all other comorbidities in our sample as autism has been known to go hand in hand with a multitude of psychiatric conditions (Joshi et al., 2013; Damiano et al., 2014). Importantly, it has been shown that depression could, for example, influence the specificity of autobiographical memories (van Vreeswijk and de Wilde, 2004; Raes et al., 2006) which could have influenced narrative coherence outcomes. Similarly, attention and concentration difficulties (ADHD) could have had a negative influence on the performance of the sequencing tasks (reaction times were measured, and the tasks were quite lengthy). This is important to consider as ADHD and autism share a large comorbidity (Stevens et al., 2016). Dyslexia would have had a negative influence on the verbal sequencing tasks specifically. Unfortunately, we did not include such information in the final analyses. Future replications are therefore warranted to take information about comorbidities into consideration.

Third, It is worth noting that concerns may be raised regarding the intelligence levels of our sample as we have tested intelligence with a non-standard intelligence test. We are aware that RPM mainly taps into analytical (*fluid*) intelligence in contrast to crystallized intelligence (Carpenter et al., 1990) and that it overestimates intelligence in some subpopulations of autism compared to standard intelligence tests (Mottron, 2004; Dawson et al., 2011). However, specifically for autism, *standard tests* might also underestimate intelligence (Dawson et al., 2007). To corroborate the intelligence scores gained from the RPM, we asked all our participants about their education level and employment details. All participants that were employed had a job that required average intelligence and all participants were well-educated.

Fourth, it could be noted that our sample size might be too small to draw firm conclusions from the data. The small sample size of *n* = 32, could have reduced the power of the current study, consequently leading to an increased probability of falsely detecting true effects (Button et al., 2013) and/or falsely accepting a null hypothesis (Shreffler and Huecker, 2022). However, compared to previous intervention studies, our sample size still seems above average. To corroborate this, we calculated the average sample size for 48 unique intervention studies reported in two meta-analyses (Lorenc et al., 2017; Benevides et al., 2020). After further excluding 10 case report studies, the average sample size was 25.21 participants for the 38 remaining intervention studies. Our sample size of 32 is higher. Furthermore, we included only 46.88% of males in the current study compared to an average of 81% males in the meta-analyses, thereby moving away from the predominantly male-based samples found in previous research. This does not, however, justify the smaller sample size and potentially reduced power in the current pilot study. Future research should include a larger sample to draw firmer conclusions, and follow-up measures to track any long-term effects.

Finally, given that the cerebellum is implicated in non-motor processes directly related to our training, such as sequencing social actions and mentalizing, future interventions could benefit from applying simultaneous cerebellar neurostimulation as well. For example, cerebellar neurostimulation could benefit the current training program by boosting neural plasticity and cognitive flexibility, leading to the replacement of maladaptive patterns of social sequencing (e.g., replacing rigid social strategies) with more adaptive ones (Van Overwalle et al., 2021). Recent studies have already revealed beneficial effects of cerebellar neurostimulation on social sequence learning (Ballard et al., 2019; Heleven et al., 2021). A recent literature overview by Dedoncker et al. (2021) advises to apply neurostimulation *during* clinical intervention as its effects seem largest when applied to an already active brain region.

## Conclusion

Even though research has identified difficulties with mentalizing and narrative coherence as core characteristics of autism, and even though neuroscience has revealed a strong causal role of the cerebellum in these processes and in the etiology of autism, interventions for autistic adults that are based on this information are still lacking. The current narrative sequencing and mentalizing training was the first to incorporate these novel neuroscientific insights to develop an intervention for this population. And even though the training is perhaps not yet optimized for clinical implementation, the current findings revealed interesting effects. Narrative coherence was improved on several dimensions, especially regarding chronological and thematic (i.e., personal reflections and evaluations) coherence. Overall coherence was improved as well. Mentalizing during sequencing was also improved on several belief conditions, while mentalizing on a more “advanced” mentalizing task without explicit sequencing did not improve. Our results suggest, for the first time, that cerebellar sequencing theories could bridge an important gap between theoretical insights and clinical implementation and highlight the need for more neuroscience-informed interventions for autistic adults. Finally, participant feedback revealed a strong need for more adult-specific training programs and revealed promising markers for the feasibility of the current program. Future research with larger samples and improved training is advised.

## Data availability statement

The original contributions presented in this study are included in the article/Supplementary material, further inquiries can be directed to the corresponding author.

## Ethics statement

The studies involving human participants were reviewed and approved by the Medical Ethical Committee of the University Hospital Brussels. The patients/participants provided their written informed consent to participate in this study.

## Author contributions

TB, FV, and EH contributed to the study conception and design. Material preparation, data collection, and analysis were performed by TB with feedback from EH. TB prepared the first draft of the manuscript. All authors commented on previous versions of the manuscript, read and approved the final manuscript.
