# Supplementary material for: A narrative sequencing and mentalizing training for adults with autism: A pilot study
Source: Front Behav Neurosci. 2022 Aug 18;16:941272. doi: 10.3389/fnbeh.2022.941272 (PMC9433774; doi:10.3389/fnbeh.2022.941272)
Supplement: Supplementary file 1 [file Data_Sheet_1.docx]

Supplementary Material

# Supplementary Data

Specifics of each session and deviations from the general steps outlined in the main article, can be found in section 4.

Information on outliers and missing data can be consulted in Supplementary Table 1.

All mean data of the sequencing tasks, including data of all scenarios (i.e., mechanical, social script, false belief, and true belief) and accuracy and reaction times, can be consulted in Supplementary Table 2. Mean data for the Narrative Coherence Coding Scheme (NaCCS) can be consulted in Supplementary Table 3. Supplementary Figure 1 provides a visual comparison between the Training group and the waiting-list Control group for NaCCS outcomes. Unless full anonymity of participants cannot be guaranteed, all other data are available from the first author upon request.

# Supplementary Tables

Supplementary Table 1. Outliers and Missing Data

| **Training Group** | | | | | |
| --- | --- | --- | --- | --- | --- |
| **Task** | **Pre (*n = 17*), Post (*n* = 14)** | **Outliers (*N*)** | **Missing data (*N*)** | **Reason for missing data** |  |
| **Verbal Sequencing Task** | Pre | 0 | 0 | No missing data | |
|  | Post | 0 | 0 | ~ | |
| **Pictorial Sequencing Task** | Pre | 0 | 0 | No missing data | |
|  | Post | 0 | 0 | ~ | |
| **NaCCS** | Pre | 1 | 0 | No missing data | |
|  | Post (same) | 0 | 0 | ~ | |
|  | Post (new) | 1 | 0 | ~ | |
| **ATT** | Pre | 0 | 0 | No missing data | |
|  | Post | 0 | 0 | ~ | |
| **Waiting-list Control Group** | | | | | |
| **Task** | **Pre (*n = 15*), Post (*n = 15*)** | **Outliers (*N*)** | **Missing data (*N*)** | **Reason for missing data** | |
| **Verbal Sequencing Task** | Pre | 0 | 1 | Emotionality + fatigue | |
|  | Post | 0 | 2 | Fatigue | |
| **Pictorial Sequencing Task** | Pre | 0 | 1 | Emotionality + fatigue | |
|  | Post | 0 | 1 | Concentration difficulties | |
| **NaCCS** | Pre | 0 | 0 | No missing data | |
|  | Post (same) | 0 | 1 | Participant found it too difficult to share stories | |
|  | Post (new) | 0 | 1 | ~ | |
| **ATT** | Pre | 0 | 2 | Recorder malfunctions | |
|  | Post | 0 | 1 | Participant did not want to continue (no specific reason provided) | |

^Note that the Training group initially started with 17 participants. 2 participants dropped from the program prior to the first session due to unexpected schoolwork. 3 participants dropped out from the training due to scheduling difficulties. From the latter, 2 were retested after completing 4 out of 6 sessions. ~ = same as above.^

Supplementary Table 2. Mean Reaction Times and Accuracy Scores for the Verbal and Pictorial Sequencing Tasks

| **Verbal Sequencing Task** | | | | | |
| --- | --- | --- | --- | --- | --- |
| **Group (Pre or Post)** | **RT, Accuracy** | **Mechanical** | **Social Script** | **True Belief** | **False Belief** |
| **Training (Pre)** | RT | 27736 (*8931*) | 25802 (*5358*) | 34790 (*8634*) | 34361 (*9873*) |
|  | RT1 | 16903 (*6173*) | 15289 (*4385*) | 21715 (*6098*) | 21755 (*6779*) |
|  | Accuracy | 5.87 (*.16*) | 5.85 (*.18*) | 5.52 (*.37*) | 5.84 (*.22*) |
| **Training (Post)** | RT | 24910 (*4141*) | 22476(*4803*) | 36098 (*9750*) | 30161 (*6599*) |
|  | RT1 | 14398 (*3652*) | 12710 (*3527*) | 20676 (*5297*) | 17731 (*4507*) |
|  | Accuracy | 5.72 (*.38*) | 5.92 (*.13*) | 5.58 (*.32*) | 5.80 (*.31*) |
| **Control (Pre)** | RT | 38508 (*21972*) | 32444 (*16670*) | 45483 (*17370*) | 46345 (*202207*) |
|  | RT1 | 12297 (*4482*) | 16597 (*6240*) | 19446 (*10580*) | 20874 (*7390*) |
|  | Accuracy | 5.44 (*.43*) | 5.82 (*.26*) | 5.24 (*.68*) | 5.16 (*.68*) |
| **Control (Post)** | RT | 33107 (*12543*) | 28915 (*8890*) | 49732 (*25753*) | 40836(*15667*) |
|  | RT1 | 20369 (*11343*) | 17248 (*7666*) | 32302 (*24951*) | 25541 (*11631*) |
|  | Accuracy | 5.56 (*.55*) | 5.81 (*.29*) | 5.23 (*.67*) | 5.61 (*.44*) |
| **Pictorial Sequencing Task** | | | | | |
| **Group (Pre or Post)** | **RT, Accuracy** | **Mechanical** | **Social Script** | **True Belief** | **False Belief** |
| **Training (Pre)** | RT | 19203 (*4934*) | 22845(*6076*) | 26867(*6129*) | 26948 (*9060*) |
|  | RT1 | 10431 (*3505*) | 12717 (*3372*) | 15591 (*3374*) | 16453 (*6997*) |
|  | Accuracy | 5.78 (*.29*) | 5.75 (*.28*) | 5.80 (*.31*) | 5.60 (*.37*) |
| **Training (Post)** | RT | 16893 (*4713*) | 21887 (*7403*) | 24542 (*5443*) | 23662 (*7942*) |
|  | RT1 | 8221 (*3701*) | 13012 (*5736*) | 14944 (*4058*) | 15192 (*5651*) |
|  | Accuracy | 5.80 (*.28*) | 5.78 (*.26*) | 5.69 (*.39*) | 5.74 (*.31*) |
| **Control (Pre)** | RT | 23287 (*5387*) | 30203 (*9566*) | 34664 (*10827*) | 34897 (*10848*) |
|  | RT1 | 12297 (*4482*) | 16597 (*6240*) | 19446 (*10580*) | 20874 (*7390*) |
|  | Accuracy | 5.72 (*.27*) | 5.80 (*.31*) | 5.76 (*.47*) | 5.37 (*.80*) |
| **Control (Post)** | RT | 19949 (*5183*) | 26917 (*10305*) | 29836 (*8579*) | 31490 (*11433*) |
|  | RT1 | 9454 (*2982*) | 14830 (*6330*) | 16701 (*7267*) | 20004 (*8221*) |
|  | Accuracy | 5.71 (*.49*) | 5.27 (*.99*) | 5.29 (*.70*) | 5.71 (*.39*) |

^RT = Reaction times in milliseconds between stimulus onset and end of trial, RT1 = Reaction times in milliseconds between stimulus onset and first response. Data is presented in the format: Mean (^*^SD^*^). Accuracy scores are the mean scores for each trial (scored between 0 and 6).^

Supplementary Table 3. Mean and Standard Deviations of NaCCS

| **Narrative Dimension** | **Training Group** | | **Control Group** | |
| --- | --- | --- | --- | --- |
|  | **Pre** | **Post (same)**  **Post (new)** | **Pre** | **Post (same)**  **Post (new)** |
|  | Negative Stories | | | |
| **Chronological** | 1.94 (*1.20*) | 2.37 (*0.70*)  2.67* (*0.47*) | 2.07 (*1.03*) | 1.74 (*1.10*)  1.48 (*1.13*) |
| **Contextual** | 1.53 (*1.01*) | 1.84 (*0.78*)  2.40* (*0.59*) | 1.80 (*0.94*) | 1.70 (*0.88)*  2.10 (0.89*)* |
| **Thematic** | 1.53 (*0.94*) | 1.86 (*1.17*)  2.07 (*0.90*) | 2.13 (*0.83*) | 1.85 (*0.64*)  2.08 (*0.80)* |
| **Overall** | 4.94 (*1.85*) | 6.07* (*1.66*)  7.14** (*1.38*) | 6.00 (*1.60*) | 5.29 (*1.43*)  5.66 (*1.62*) |
|  | Positive stories | | | |
| **Chronological** | 1.82 (*1.24*) | 2.66* (*0.53*)  2.57 (*0.53*) | 1.67 (*1.11*) | 1.47 (*1.06*)  1.12 (*1.05*) |
| **Contextual** | 2.06 (*0.97*) | 2.03 (*0.75*)  2.37 (*0.78*) | 1.80 (*1.01*) | 1.65 (*0.97*)  1.82 (*1.09*) |
| **Thematic** | 1.29 (*0.58*) | 2.27** (*0.75*)  2.07* (*0.97)* | 2.47 (*0.74)* | 2.09 (*0.80*)  2.01 (*0.76*) |
| **Overall** | 5.24 (*1.79*) | 6.84* (*1.23*)  7.01* (*1.65*) | 5.93 (*1.62*) | 5.21 (*1.57*)  4.94 (*1.58*) |

^*^ *^p^* ^< .05, **^ *^p^* ^<^ *^.001^*

# Supplementary Figure


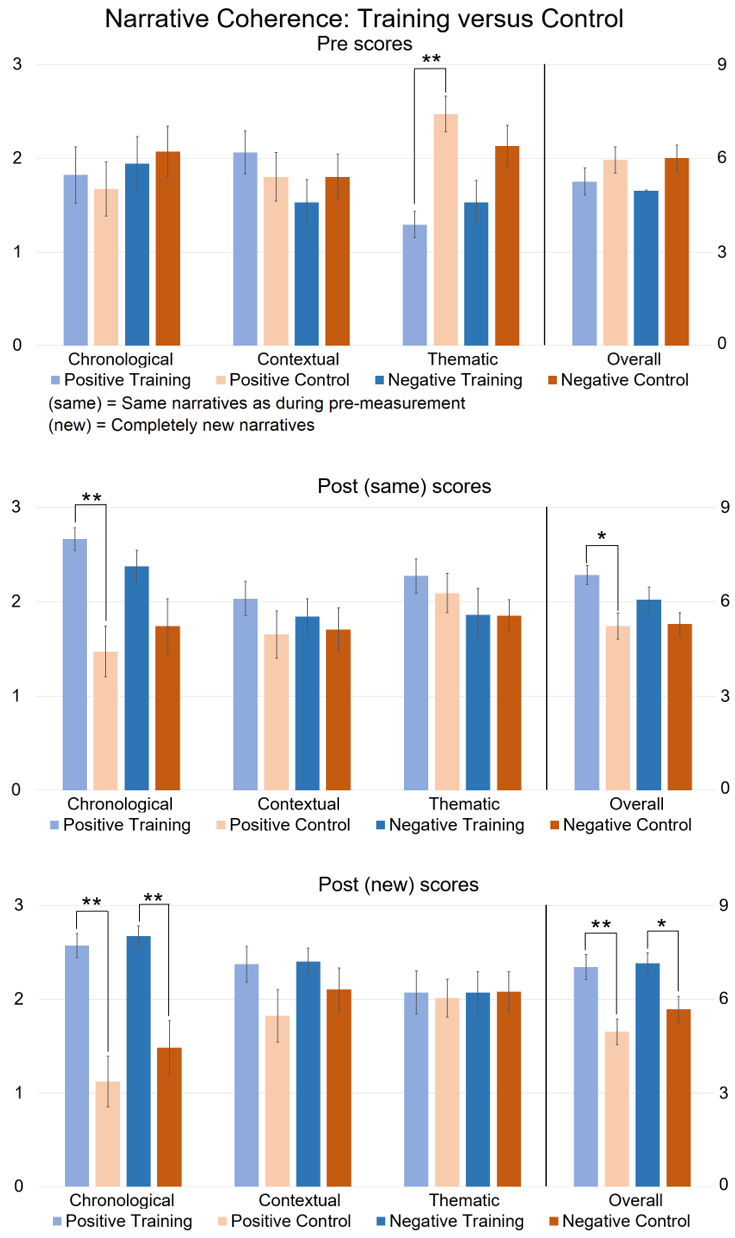


Supplementary Figure 1. Mean narrative coherence scores comparing the Training group with the Control group. The 0 – 3 scale on the left refers to the three distinct coherence dimensions, while the 0 – 9 scale on the right refers to the overall coherence. The post-measurement refers to narratives that are the “same” or “new” compared to the pre-measurement. Error bars represent the Standard Error of Means (SEM).

# Sessions in more detail

### Session 1: Retelling stories with visual aids

This session focused on narrative structure. First, the investigator described the main parts of a narrative (beginning, middle, and end). Second, the mini lesson focused on story grammar elements that were associated with icons and shared via a PowerPoint presentation. Participants were aided in their retelling by sharing the icons in the correct sequence. The homework assignment consisted of identifying story grammar elements.

### Session 2: Retelling stories with visual aids and transition words

This session included a mini lesson focused on transition words to causally and temporally link story grammar elements. Participants were asked to brainstorm about transition words that could be used to link the story grammar elements. Stories were compared with and without transition words, and participants were asked to reflect on the coherence of these examples. During retelling, participants could use chronologically sequenced icons and a list of transition words that were shared on a PowerPoint presentation. In the second homework assignment, participants read two narratives and were required to fill in blank lines with adequate transition words.

### Session 3: Retelling stories without visual aids

The mini lesson in this third session elucidated the concept of mentalizing. Hands-on tools were provided to help reflect on own mental states, as well as to help understand others’ mental states. During retelling, participants had to retell the narrative without visual aids but were strongly encouraged to keep the grammar elements in mind. In the third homework assignment, participants had to read three narratives and answer mental state questions.

### Session 4: Generating stories with visual aids and prompts

Session 4 marked the transition from purely retelling prewritten narratives to generating self-experienced narratives. The last mini lesson summarized the main topics of the previous sessions. To generate narratives, a PowerPoint slide was shared that contained all icons in chronological order. Next to each icon, prompts were provided to aid the participant in telling a story (e.g., Setting: ‘Library’), and to ensure a gradual transition from purely retelling to generating narratives so that motivation would not diminish, or practice would not immediately be too challenging. One participant generated a narrative using the icons and the accompanying prompts while another participant retold this narrative. The homework assignment was preparatory for session 5, as discussed below.

### Session 5: Generating stories through home preparations

Participants were required to generate personally experienced narratives through the homework preparations which consisted of four parts: (1) story breakdown into beginning, middle, and end, (2) story grammar element identification, (3) adding transition words, and (4) writing the story down. During storytelling, participants were allowed to use the pages containing the story grammar elements. Participants’ performance was videotaped (see session 6). Participants were required to ask questions themselves. The investigator helped by a priori sending participants a document with example questions. The last homework assignment consisted of two scrambled narratives which participants had to place in the correct chronological order.

### Session 6: Spontaneously generating stories

Participants were encouraged to reflect on their storytelling abilities through watching and discussing their videotaped performance. The investigator provided feedback and invited the participants to critically reflect by using metacognitive strategies (e.g., “Do you believe that your story was adequately structured?”). Next, spontaneous storytelling was instigated by minimal prompts such as, “what did you do this weekend?”
